# ABCs or 123s? The independent contributions of literacy and numeracy skills on health task performance among older adults

**DOI:** 10.1016/j.pec.2015.04.007

**Published:** 2015-08

**Authors:** Samuel G. Smith, Laura M. Curtis, Rachel O’Conor, Alex D. Federman, Michael S. Wolf

**Affiliations:** a Health Literacy and Learning Program, Division of General Internal Medicine and Geriatrics, Northwestern University, Chicago, USA; b Wolfson Institute of Preventive Medicine, Queen Mary University of London, London, UK; c Division of General Internal Medicine, Mount Sinai School of Medicine, New York, USA; d Department of Learning Sciences, Northwestern University, Chicago, USA

**Keywords:** Health literacy, Numeracy, Health communication, Patient education

## Abstract

**Objective:**

To investigate the relationship between literacy and numeracy and their association with health task performance.

**Methods:**

Older adults (*n* = 304) completed commonly used measures of literacy and numeracy. Single factor literacy and numeracy scores were calculated and used to predict performance on an established set of health self-management tasks, including: (i) responding to spoken information; (ii) comprehension of print and (iii) multimedia information; and (iv) organizing and dosing medication. Total and sub-scale scores were calculated.

**Results:**

Literacy and numeracy measures were highly correlated (*r*s = 0.68; *p*s < 0.001). In multivariable models adjusted for age, gender, race, education, and comorbidity, lower literacy (*β* = 0.44, *p* < 0.001) and numeracy (*β* = 0.44, *p* < 0.001) were independently associated with worse overall task performance and all sub-scales (literacy range, *β* = 0.23–0.45, *p*s < 0.001; numeracy range, *β* = 0.31–0.41, *p*s < 0.001). Multivariable analyses with both constructs entered explained more variance in overall health task performance compared with separate literacy and numeracy models (8.2% and 10% respectively, *p*s < 0.001).

**Conclusion:**

Literacy and numeracy were highly correlated, but independent predictors of health task performance. These skill sets are complementary and both are important for health self-management.

**Practice implications:**

Self-management interventions may be more effective if they consider both literacy and numeracy skills rather than focusing on one specific ability.

## Highlights

• Literacy and numeracy are highly correlated.
• In multivariable analysis, literacy and numeracy were independent predictors of health task performance.
• Literacy and numeracy are complementary skills and both are important for health self-management.

## 1 Introduction

Limited health literacy is associated with adverse health outcomes including worse functional health status [1], [2], [3], [4], greater hospitalization rates [5], [6], [7], and increased all-cause mortality [8], [9], [10]. The strongest evidence exists for a relationship between health literacy and the performance of health tasks; including the interpretation of health text and labels [11], [12], safe use of medication [13], [14], [15], and preventive screenings [16], [17]. The construct has been defined by the Institute of Medicine as: ‘the degree to which individuals have the capacity to obtain, process, and understand basic health information and services needed to make appropriate health decisions’ [18].

There is debate surrounding what the construct represents and how to measure it [2], [19]. The World Health Organization's definition put forth by Nutbeam states that functional literacy is the ability to read and write in a medical context [20]. However, this neglects the important contribution of numeracy, which is often needed to perform and execute basic health tasks. As a very basic example, when patients prepare for a colonoscopy they must be able to read and comprehend the preparatory instructions, but also calculate the dose of laxative and time it appropriately with food [11]. Subsequent publications from Nutbeam have included numeracy skills within the definition of health literacy [21], but it is clear that empirical data are needed to further understand the role of numeracy within the concept of health literacy.

Multiple definitions of numeracy exist [22]. Here, we shall use the broad definition used by Reyna and colleagues, ‘the ability to understand and use numbers’. Numerical health data are rarely presented in isolation, and are instead often embedded within qualitative text. For example, disease incidence data in patient information leaflets [23], or drug side-effects on medicinal labels [12]. The close interaction between the concepts has led some investigators to refer to numeracy as ‘quantitative literacy’ [24], [25]. This conceptual distinction further emphasizes the need for empirical data in this area.

Within the most comprehensive systematic review of health literacy research, literacy and numeracy were examined separately [26]. Berkman and colleagues reported that no firm conclusions could be made about the association between numeracy and most of the health outcomes investigated. However, several reviews and theoretical frameworks have argued for the importance of numeracy in specific medical decision-making tasks [22], [27], [28], [29]. For example, low numeracy has been associated with poorer risk estimation [30], greater susceptibility to biases and framing effects [31], [32] and less trust in numerical information [32]. While these cognitive mechanisms may not be considered clinical outcomes, they are pathways through which numeracy can influence health and wellbeing.

The issue is further complicated by the inclusion of numeracy components within health literacy measures. Several health literacy measures exist, but the most common are the Rapid Estimate of Adult Literacy in Medicine (REALM) [33], the Test of Functional Health Literacy in Adults (TOFHLA) [34], and the Newest Vital Sign (NVS) [35]. While the REALM reflects the ability to read in a medical situation, success on the TOFHLA and NVS depends on both numeracy and literacy skills. Research examining the unique and combined contribution of literacy and numeracy to health outcomes is lacking.

Intervention strategies to mitigate issues with literacy are likely to be different from those addressing numeracy problems. For example, where a patient with poor literacy is experiencing difficulty, a response may be to simplify the text [36], [37], [38] or deconstruct the task to increase the ease of completion [39], [40]. In contrast, if numeracy is the issue, effective strategies may include altering the presentation of numerical data [41], [42] or providing decisional support [31], [43], [44]. To advance health literacy research and intervention strategies, further investigation is required into the independent contributions of literacy and numerical abilities on health self-management.

Existing studies have examined the role of both literacy and numeracy measures on health-related outcomes. For example, in a cohort of inpatients hospitalized with acute coronary syndromes and/or acute decompensated heart failure, higher subjective numeracy and higher objective health literacy were independently associated with lower odds of making post-discharge medication errors [45]. In a cohort of acute heart failure patients, low subjective numeracy was associated with having an unplanned return to hospital within 30 days of discharge, but subjective literacy was not [46]. The independent contribution of both measures was not addressed in the same statistical model. Finally, among a sample of type 1 and 2 diabetics, objective numeracy was associated with self-efficacy for diabetes self-management, but there was no association with objective health literacy [47]. These inconsistent findings, the reliance on subjective measures, and the heterogeneous outcomes assessed suggest further research is warranted.

The literacy and cognitive function among older adults (LitCog) cohort provides an opportunity to investigate this area as several measures of objective literacy and numeracy were recorded at baseline [48]. This sample is particularly relevant as health literacy has been shown to decrease with age, while the likelihood of engaging in health self-management tasks increases [49]. Using this sample of older American adults, we investigated the relationship between literacy and numeracy as well as their unique and combined association with performance on an established set of health self-management tasks.

## 2 Methods

### 2.1 Sample

A full description of the recruitment procedures and methods has been published elsewhere [48]. Briefly, the LitCog sample was recruited between August 2008 through October 2010 from one academic primary care clinic at Northwestern Memorial Hospital and four federally qualified health centers (FQHC's) in Chicago. Participants were eligible if they: (1) spoke English (2) were between the ages 55–74, and (3) lacked any hearing, visual or cognitive impairment. Northwestern University's Institutional Review Board approved the project. A total of 828 participants were enrolled at baseline. For this sub-study, the first 321 participants enrolled onto LitCog were asked to complete an additional numeracy scale. Seventeen people refused to complete at least one literacy or numeracy assessment, providing a total sample of 304 participants to be included here.

### 2.2 Procedure

In-person structured interviews were held in a private room at Northwestern Memorial Hospital or at one of the four FQHCs. Two sessions were undertaken lasting 2.5 hours and spaced 7–10 days apart. Trained interviewers administered a series of assessments and questionnaires related to literacy, numeracy, performance on common health tasks, and participant characteristics. Prior to the interview, prospective participants were told that the study includes people who have been seen at the clinics in the Access Community Health Network and their doctor agreed that they were eligible to take part. They were informed that the overall aim of LitCog was to aid the creation of better health learning tools to assist patients with their day-to-day healthcare. Free parking or travel reimbursement was provided to encourage participation.

#### 2.2.1 Literacy assessments

A single factor score for literacy was computed using three different assessments. This was done to provide one factor that could represent the multitude of skills that the following measures assess under the umbrella term ‘literacy’. The Rapid Estimate of Adult Learning in Medicine (REALM) assesses correct pronunciation of a list of 66 words related to healthcare [33]. The American version of the National Adult Reading Test (AM-NART) was also used and involves reading a list of 45 non-medical words [50]. For the REALM and the AM-NART, the interviewer records correct pronunciation. The final literacy assessment was the reading component of the Test of Functional Health Literacy in Adults (TOFHLA-R) [34]. This assessment uses the cloze procedure, whereby every fifth to seventh word in a passage of increasingly difficult health-related text is missing. Participants are required to fill in missing words using multiple choice response options. One point is awarded for each correct selection from the multiple choices, and score are transformed to range from 0 to 50. The TOFHLA-R does not measure pronunciation, but instead assesses medical vocabulary knowledge and the ability to quickly manipulate sentences to ensure comprehension.

#### 2.2.2 Numeracy assessments

A single factor score for numeracy was computed using three different assessments, again because each numeracy measure is likely to represent a different skill set. All instructions for the numeracy assessments were read to participants verbally, thereby limiting the need for literacy in these tasks. The Newest Vital Sign requires participants to review a nutrition label and answer items based on how they would interpret and react to the information [35]. To successfully answer the questions, numerical calculations are required. The score range was 0–6. The Lipkus Numeracy Scale was also recorded [51]. This requires participants to complete 11 objective numeracy items that increase in difficulty. An example item is: ‘Which of the following numbers represents the biggest risk of getting a disease? 1 in 100, 1 in 1000, 1 in 10.’ The score range was 0–11. The Lipkus assessment generally involves the ability to understand risk and manipulate probabilities. Finally, the numeracy component of the TOFHLA was recorded (TOFHLA-N). This assessment involves reviewing several patient instruction cards such as appointment slips and example results from a medical test. To answer questions, participants are required to perform mental arithmetic such as date subtraction and dosing calculations. The score range was 0–50.

#### 2.2.3 Health task scenarios

The Comprehensive Health Activities Scale (CHAS) was developed as an outcome for the LitCog cohort. It has been described in detail elsewhere [52]. Briefly, it requires participants to engage and respond to tasks that they may have to perform to manage their health in everyday life. Participants are shown 10 hypothetical health scenarios in a variety of different formats (print, video, verbal and artifacts [e.g. pill bottles]). After each scenario, they are asked to answer a series of questions. Scores were standardized to range from 0 to 100, and item subscales created. These included recalling spoken information (7 items); comprehension of print information (18 items); recalling multimedia information (7 items); and organizing and dosing medication (13 items). Higher scores correspond to better performance. See Table 1 for a description of the tasks.

#### 2.2.4 Participant characteristics

The following participant characteristics were recorded: age (55–64, 65–74), gender, race (White, African American, Other), and education (≤High school, some college or technical school, college graduate, graduate degree) were recorded. The following comorbidities were also self-reported: arthritis; asthma; bronchitis or emphysema; cancer; coronary heart disease; depression; diabetes; heart failure; and hypertension. These data were *categorized as* 0, 1 and 2 or more comorbidities.

### 2.3 Power calculator

The sample size for LitCog was based on the primary outcomes of the main LitCog analysis [48]. Sensitivity power analyses for this study assuming *α* = 0.05, 90% power, and 7 predictors in a multivariable regression analysis suggested a sample of 304 participants would be sufficient to detect a small effect size of 0.06 [53].

### 2.4 Analysis

Participant characteristics were presented using descriptive statistics. Factor scores for literacy (REALM, AM-NART, TOFHLA-R) and numeracy (NVS, Lipkus, TOFHLA-N) were computed to reduce the skills to two measures for subsequent regression models and avoid multicollinearity. Maximum likelihood estimation was used because it is a valid and statistically efficient way of creating single factor solutions when assessing multiple theoretically similar constructs that are strongly interrelated [54]. Literacy and numeracy represent vast concepts composed of multiple skills [25]. As described in the measures section, the assessments shown are commonly used but when collected individually they are likely to offer a somewhat crude interpretation and single perspective around the underlying constructs of ‘literacy’ and ‘numeracy’. For example, the TOFHLA-N assesses date calculation, addition and subtraction, while the Lipkus assessment focuses on risk and comprehension of probabilities. Using a single factor score composed of multiple measures offers a more robust and precise measure of the latent skill and does not rely on one individual measure. The association between participant characteristics and the literacy and numeracy scores were done using Analysis of Variance. Pearson's correlations compared the associations between the literacy and numeracy measures. A series of multivariable linear regression models examined the relationship between literacy, numeracy and health task performance. Standardized regression coefficients (*β*) are reported throughout. The multivariable models were adjusted for age, gender, race, education, and comorbidity. Models were run entering literacy (Model 1) or numeracy (Model 2) to establish the extent to which they predicted health task performance (total score and all sub-scales). Model 3 then entered both literacy and numeracy to examine their combined effects on health task performance. F-tests determined if the variance explained by each of the models (*R*^2^) significantly changed with the addition of the other variable (i.e. Model 1 vs. Model 3 and Model 2 vs. Model 3). Statistical significance was set at *p* < 0.05 for all analyses. Analyses were performed in SPSS version 22.0.

## 3 Results

### 3.1 Participant characteristics

As shown in Table 2, participants were mostly female (74.7%), White (59.3%) or African American (34.7%) and had either a college degree (20.1%) or a graduate degree (37.8%). The average age of the sample was 63.2 years (SD = 5.4), ranging from 55 to 74 years. Participants had between 0 and 8 comorbidities, with an average of 1.7 (SD = 1.3).

The average literacy score on the single-item factor was 0.19 (SD = 0.70) and it ranged from −3.8 (low literacy) to 0.75 (high literacy). The average numeracy score was 0.00 (SD = 0.89) and it ranged from −2.27 (low numeracy) to 1.71 (high numeracy). Higher scores indicated better performance. Literacy scores were associated with race (*p* < 0.001), and African American participants scored lowest (Table 2). Literacy decreased with less education (*p* < 0.001) and was lowest in those with more chronic conditions (*p* = 0.002). Similar observations were apparent for associations between numeracy and comorbidities (*p* < 0.001), education (*p* < 0.001) and race (*p* < 0.001). Numeracy was also lower among women (*p* = 0.008) and older participants (*p* < 0.001).

### 3.2 Associations between literacy and numeracy measures

As shown in Table 4, participants scored highly on the REALM (*M* = 61.5 out of 66, SD = 8.8) and the TOFHLA-R (*M* = 45.6 out of 50, SD = 7.0) (Table 3). Scores were moderate on the AM-NART (*M* = 29.6 out of 45, SD = 11.2) and all numeracy measures (Lipkus *M* = 5.8 out of 11, SD = 2.6; NVS *M* = 3.6 out of 6, SD = 2.1; and TOFHLA-N *M* = 35.3 out of 50, SD = 15.1). As shown in Table 3, all measures of literacy and numeracy were strongly correlated (rs range = 0.39 to 0.60, *p* < 0.001 for all).

### 3.3 Associations between literacy, numeracy and health task performance

#### 3.3.1 Overall health task performance

Health task scores ranged from 6.3 to 97.8 (*M* = 67.2 out of 100, SD = 18.9). After adjusting for age, gender, race, education, and comorbidity, higher literacy scores predicted better health task performance (Model 1; *β* = 0.65; *p* < 0.001) (Table 5). When numeracy was entered into a separate model, higher numeracy was associated with better health task performance (Model 2; *β* = 0.68; *p* < 0.001). When literacy and numeracy were included concurrently, both were significant predictors of performance (Model 3; *β* = 0.44 and *β* = 0.44, respectively, *p*s < 0.001).

To test whether including both literacy and numeracy significantly improved the explanatory power of the models, the *R*^2^ change statistic was observed. There were significant differences in variance explained between Model **1** and Model **3** (*R*^2^ change = 0.082; (*F*(1, 282 change = 84.96, *p* < 0.001) and Model **2** and Model **3** (*R*^2^ change = 0.10; (*F*(1, 282) change = 99.33, *p* < 0.001) indicating models containing literacy and numeracy are better than those considering each variable alone.

#### 3.3.2 Health task performance sub-scales

For comparison, the scores on each of the sub-scales were standardized to range from 0 to 100. As shown in Table 3, average scores were: comprehension of print information (*M* = 72.5, SD = 20.9); organizing and dosing medication (*M* = 68.1, SD = 20.4); responding to spoken instructions (*M* = 73.3, SD = 21.6); and recalling multimedia information (*M* = 48.3, SD = 27.7). This indicates that participants found tasks related to recalling multimedia information more difficult.

For all sub-scales, the importance of both literacy and numeracy was similar to the pattern observed for the overall scale (see Table 5). Higher literacy (Model 1s, *β* range = 0.39–0.63, all *p*s < 0.001) and higher numeracy (Model 2s, *β* = 0.45–0.65, all *p*s < 0.001) were associated with better performance on all tasks. When entered together, literacy and numeracy were both significantly associated with performance on all tasks (literacy range, *β* = 0.23–0.45, all *p*s < 0.001; numeracy range, *β* = 0.31–0.41, all *p*s < 0.001). Significantly more variance was explained in models including literacy and numeracy together compared with each alone (data not shown).

## 4 Discussion and conclusion

### 4.1 Discussion

In this sample of older American adults, higher literacy and numeracy skills were independent and comparable predictors of successful health task performance. This finding was maintained when both were entered into the same model, and after controlling for participant characteristics such as age, gender, race, education, and comorbidity. Entering both skills into the model in tandem led to a significant increase in the amount of variance explained in health task performance. Considering literacy or numeracy skills in the performance of routine health tasks may be important, but researchers and clinicians should be mindful of both skills to maximize the chances of successful intervention.

These findings support our earlier work and that of others demonstrating the importance of health literacy in common self-management health tasks, such as recalling spoken instructions and comprehending written information [11], [26], [48]. However, using existing data from the LitCog sample allowed us to investigate the distinct and combined role of literacy and numeracy skills in health task performance. Investigations in this area are needed to facilitate the development of health literacy definitions, measures and intervention strategies.

The IOM definition outlines health literacy as a skills-based construct that facilitates effective health self-management [18]. In support of this, we demonstrated that a large proportion of variance in the public's ability to perform simple tasks was explained by a patient's basic skill level. Our findings however go beyond this observation and show that this capacity is not simply the ability to read in a medical context, but also aptitude in numerical processing and calculation; both are necessarily included within the larger construct of ‘health literacy’ [18]. While other ‘mindset’ factors such as patient activation are likely to be important when engaging in health self-management [2], [55], these data demonstrate the predictive power of these common assessments of literacy and numeracy. Health literacy definitions should continue to include both skills within their definitions, and studies should recognize the complementary nature of these skills rather than include only one in isolation when investigating performance on common healthcare tasks [19].

Our findings also have implications for health literacy measurement by demonstrating the importance of using assessments that include tests of both literacy and numeracy skills. Studies using literacy assessments such as the REALM, which served as the foundation of the health literacy field for decades, may need to be complemented by numeracy scales to ensure all elements of health literacy are captured. Researchers developing new health literacy measures may wish to consider taking the approach of the TOFHLA by offering two separate sub-scales that reflect literacy and numeracy abilities.

Finally, it was noticeable that average performance scores on these simple tasks were well below the maximum. This was particularly striking given the high levels of education reported in our sample, where nearly 60% had a college degree or more. This is higher than city-wide estimates for Chicago, where approximately 35% of adults are reported to have a Bachelor's degree or higher [56]. Health task performance in less educated samples may therefore be even poorer, and clinicians should be aware of this when discussing health self-management with patients. This was particularly evident for the multimedia tasks, where on average participants correctly responded to less than 50% of the items. This finding could be reflective of the older sample who may be less familiar with these technologies [57], [58]. However, it may also reflect the relative difficulty of processing, remembering and recalling verbal information that is presented only once compared with the visual tasks that could be referred to throughout.

This study had limitations. The outcome measure used in the study was a series of hypothetical tasks confined to a laboratory setting. Although every effort was made to ensure the validity of the scenarios, participants may have reacted differently when performing the tasks and making decisions in everyday life. The sample was also older, predominantly White and reported high levels of education and health literacy which may limit the generalizability of the findings beyond this cohort. There were particularly obvious ceiling effects on the TOFHLA-R and REALM assessments and therefore careful consideration should be given when using these assessments among older adults. Visual acuity was not controlled for in these analyses, and so it is possible that a proportion of the variance attributed to literacy and numeracy was actually due to problems with vision. Finally, these observations were cross-sectional which limits inferences about causality.

### 4.2 Conclusion

In this sample of older American adults, literacy and numeracy skills were independent and comparable predictors of successful health task performance. Considering both abilities together significantly increased the amount of variance explained. These findings suggest that literacy and numeracy should be considered in health literacy measurement and in future intervention strategies.

### 4.3 Practice implications

A systematic review of complex health literacy interventions demonstrated that all 15 identified trials focused on literacy rather than numeracy [59]. These findings suggest more attention should be focused on the numerical aspects of health task performance if health literacy disparities are to be reduced. Approaches to address literacy and numeracy deficits may be conceptually different. Our findings suggest that the basic approach of simplifying language (literacy; [36], [37], [38]) or altering the presentation of numerical data (numeracy; [41]) may not be sufficient in isolation. Researchers and clinicians in the health literacy field should use multifaceted intervention strategies that consider both literacy and numeracy skills in order to optimize health task performance.

## Conflict of interest statement

The authors report no conflicts of interest.

## Funding

Research reported in this publication was supported by the National Institute of Aging of the National Institutes of Health under award number R01AG030611. The content is solely the responsibility of the authors and does not necessarily represent the official views of the National Institutes of Health. Dr. Smith is currently supported by a Cancer Research UK Postdoctoral Fellowship (C42785/A17965).

## Figures and Tables

**Table 1:** Description of health scenarios.

Information presentation: task	Description
Print documents	
Consent to a procedure	Read a consent form given before an angiography and exhibit understanding of the procedure, potential complications, and physician responsibilities
Monitor blood sugar	Calculate and interpret numeric information from a chart listing 7 days of recorded blood sugar levels before and after meals for a diabetic patient
Prepare for a procedure	Review instructions for colonoscopy preparation, and demonstrate comprehension of patient duties prior to the procedure
Choose a facility	Examine written text about pressure sore prevention, a chart comparing prevention at two nursing homes, and a map in order to select the best facility
Monitor condition	Review and demonstrate understanding of a sheet about heart failure symptoms, monitoring, and self-care activities, as well as a calendar indicating weight and swelling status
Medication bottles	
Manage prescription medications	Review prescription bottles from two hypothetical prescription medication regimens; pronounce the names of the medications, make inferences on usage, and dose both regimens over a 24-hour period using a medication box
Spoken instructions	
Understand new diagnosis	Receive oral instructions from a physician regarding a diagnosis and course of treatment for GERD; answer questions to assess immediate and delayed recall about self-management
Recall home care instructions	Listen to wound care instructions for a laceration upon discharge from the emergency department; recall information about follow-up appointments, frequency of dressing change, and signs of infection
Multimedia video	
Recall symptom prevention information	Watch a video clip on identifying, monitoring, and controlling asthma triggers; recall information immediately following the video and at the end of the interview

**Table 2:** Participant characteristics (*n* = 304).

Characteristic	*n* (%)	Literacy		Numeracy	
Mean (SD)	*p* value	Mean (SD)	*p* value
Age					
55–64	194 (63.8)	0.25 (0.60)	0.057	0.13 (0.91)	0.001
65–74	110 (36.2)	0.87 (0.84)		−0.23 (0.82)
Gender					
Male	77 (25.3)	0.17 (0.71)	0.808	0.23 (0.91)	0.008
Female	227 (74.7)	0.19 (0.70)		−0.08 (0.87)
Race (%)					
African American	104 (34.7)	−.34 (0.90)	<0.001	−0.67 (0.82)	<0.001
White	178 (59.3)	0.49 (0.32)		0.41 (0.68)
Other	18 (6.0)	0.19 (0.71)		−0.35 (0.77)
Education					
≤High school	60 (19.7)	−0.58 (1.05)	<0.001	−0.78 (0.86)	<0.001
Some college/technical school	68 (22.4)	0.20 (0.49)		−0.29 (0.80)
College graduate	61 (20.1)	0.32 (0.47)		0.12 (0.75)
Graduate degree	115 (37.8)	0.50 (0.31)		0.51 (0.64)
Comorbidities					
0	51 (16.8)	0.38 (0.39)	0.002	0.29 (0.75)	<0.001
1	92 (30.3)	0.31 (0.62)		0.18 (0.79)
2+	161 (53.0)	0.06 (0.80)		−0.19 (0.94)

**Table 3:** Literacy, numeracy and health task scores.

	Mean (SD)
Performance on literacy measures	
REALM (0–66)	61.5 (8.8)
NART (0–45)	29.6 (11.2)
TOFHLA-R (0–50)	45.6 (7.0)
Performance on numeracy measures	
Lipkus (0–11)	5.8 (2.6)
NVS (0–6)	3.6 (2.1)
TOFHLA-N (0–50)	35.3 (8.0)
Health task scores	
Total scale	67.2 (18.9)
Comprehension of print information	72.5 (20.9)
Organizing and dosing medication	68.1 (20.4)
Responding to spoken instruction	73.3 (21.6)
Recalling multimedia information	48.3 (27.7)

**Table 4:** Associations between literacy and numeracy assessments (*n* = 304).

**Assessment**	REALM	NART	TOFHLA-R	Literacy	Lipkus	NVS	TOFHLA-N	Numeracy
REALM	N/A							
NART	0.73	N/A						
TOFHLA-R	0.75	0.72	N/A					
**Literacy (factor)**	**0.93**	**0.89**	**0.90**	**N/A**				
Lipkus	0.49	0.63	0.51	0.58	N/A			
NVS	0.48	0.69	0.59	0.64	0.61	N/A		
TOFHLA-N	0.39	0.48	0.46	0.46	0.53	0.49	N/A	
**Numeracy (factor)**	**0.54**	**0.73**	**0.62**	**0.68**	**0.90**	**0.84**	**0.73**	N/A

^*^All correlations significant at *p* < 0.001.

**Table 5:** The impacts of literacy and numeracy skills on health task performance.

	Model I literacy	Model II numeracy	Model III literacy + numeracy
Measure			
Overall health task performance			
Literacy factor score	0.65***	–	0.44***
Numeracy factor score	–	0.68***	0.44***
	*R*^2^ = 0.65	*R*^2^ = 0.65	*R*^2^ = 0.73
Printed documents			
Literacy factor score	0.63***	–	0.45***
Numeracy factor score	–	0.65***	.41***
	*R*^2^ = 0.60	*R*^2^ = 0.59	*R*^2^ = 0.67
Dosing medicines			
Literacy factor score	0.56***	–	0.39***
Numeracy factor score	–	0.56***	0.37***
	*R*^2^ = 0.47	*R*^2^ = 0.45	*R*^2^ = 0.53
Spoken instructions			
Literacy factor score	0.39***	–	0.26***
Numeracy factor score	–	0.45***	0.31***
	*R*^2^ = 0.34	*R*^2^ = 0.36	*R*^2^ = 0.38
Multimedia			
Literacy factor score	0.42***	–	0.23***
Numeracy factor score	–	0.51***	0.40***
	*R*^2^ = 0.35	*R*^2^ = 0.39	*R*^2^ = 0.42

Models are adjusted for age, gender, race, education, and comorbidity.

*** *p* < 0.001.
